# 2153: Innovative 3D printed intravaginal rings for contraception and HIV prevention

**DOI:** 10.1017/cts.2017.23

**Published:** 2018-05-10

**Authors:** Rahima Benhabbour, Rima Janusziewicz, Sue J. Mecham, Roopali Shrivastava, Gayane Paravyan

## Abstract

OBJECTIVES/SPECIFIC AIMS: The long-term goal of this project is to develop a cost effective 3D printed multipurpose intravaginal ring (IVR) to prevent against unintended pregnancies and infectious diseases.Our goal is to develop a female-controlled method for prevention using innovative IVRs. METHODS/STUDY POPULATION: In vitro and in vivo characterization. RESULTS/ANTICIPATED RESULTS: Controlled and fine-tuned release kinetics 100% drug release from 3D printed IVRs compared with 10%–15% with traditional injection molded IVRs cost-effective engineering of multipurpose IVR with CLIP 3D printing technology. DISCUSSION/SIGNIFICANCE OF IMPACT: If successful, this project will revolutionize the engineering of IVRs and will have a global impact on human health. Not only we will help save millions of women around the world but also millions of children that are infected by their HIV-positive mothers through gestation or breast feeding.

